# Multi-party open-ended conversation with a social robot 

**DOI:** 10.3389/frobt.2026.1766383

**Published:** 2026-04-15

**Authors:** Giulio Antonio Abbo, Maria Jose Pinto-Bernal, Martijn Catrycke, Tony Belpaeme

**Affiliations:** IDLab-AIRO, Ghent University – imec, Ghent, Belgium

**Keywords:** conversational agent, furhat, human-robot interaction, large language model, multi-party conversations

## Abstract

Multi-party open-ended conversation remains a major challenge in human–robot interaction, particularly when robots must recognise speakers, allocate turns, and respond coherently under overlapping or rapidly shifting dialogue. This paper presents a multi-party conversational system that combines multimodal perception (voice direction of arrival, speaker diarisation, face recognition) with a large language model for response generation. Implemented on the Furhat robot, the system was evaluated with 30 participants across two scenarios: (i) parallel, separate conversations and (ii) shared group discussion. Results show that the system maintains coherent and engaging conversations, achieving high addressee accuracy in parallel settings (92.6%) and strong face recognition reliability (80–94%) . Participants reported clear social presence and positive engagement, although technical barriers such as audio-based speaker recognition errors and response latency affected the fluidity of group interactions. The results highlight both the promise and limitations of LLM-based multi-party interaction and outline concrete directions for improving multimodal cue integration and responsiveness in future social robots.

## 1 Introduction

Rapid advances in both hardware and conversational AI have accelerated progress towards social robots capable of engaging in natural, fluid conversation with humans in a natural and intuitive way (Obaigbena et al., 2024; Schöbel et al., 2024). As a result, robots are increasingly deployed in education (Belpaeme et al., 2018; Verhelst et al., 2024), healthcare and therapy (Kyrarini et al., 2021; Cifuentes et al., 2020), as well as for companion roles (Broadbent et al., 2023; Giudici et al., 2024), where expectations for rich social and conversational behaviour continue to grow. At the same time, embodied conversational agents have achieved notable improvements in gesture generation, expressiveness, and user engagement (Wolfert et al., 2022; Aneja et al., 2021; Gibson, 2000). However, this progress has not translated equally across interaction types. While dyadic human-robot conversation has seen substantial advancements, open-ended multi-party interaction, defined as spoken interaction involving more than two participants, remains a challenge, especially when the robot must coordinate naturally with several users at once.

Multi-party interaction introduces complexities that go far beyond a dyadic conversation. Robots must identify who is speaking, distinguish overlapping utterances, determine the intended addressee, and manage turn-taking within rapidly shifting conversational dynamics (Addlesee et al., 2024; Foster et al., 2012; Moujahid et al., 2022; Murali et al., 2023; Paetzel-Prüsmann and Kennedy, 2023; Richter et al., 2016; Żarkowski, 2019). These requirements challenge perception, timing, dialogue management, and real-time adaptation. Even with recent advances in large language models (LLMs), most existing systems manage these situations by constraining turn-taking or limiting user behaviour, which restricts the naturalness and realism of the interaction.

While recent progress in LLMs has greatly strengthened the conversational abilities of social robots, enabling more flexible, context-aware, and human-like dialogue (Chang et al., 2024), these advances have been demonstrated primarily in controlled, single-speaker settings. Multi-party conversation remains mostly absent from LLM research and deployment, leaving open the question of whether these models can reliably track multiple interlocutors, maintain distinct conversational threads, or adapt to disruptions such as rapid turn switches and fragmented discourse.

Our work contributes towards this gap by exploring how multimodal perception and LLM-driven dialogue generation can support multi-party interaction. We present a conversational architecture that integrates voice direction arrival (the direction from which the voice arrives at the microphone), speaker diarisation, and face tracking with an LLM-based dialogue manager to enable open-ended exchanges between a robot and two users interacting at the same time. The system was implemented on the Furhat social robot (Al Moubayed et al., 2012), leveraging its expressive face projection and microphone array for multimodal perception.

To evaluate the system under different conversational dynamics, we conducted a controlled multi-user evaluation in which two participants interacted freely with the robot in an open-ended conversational setting. This approach was necessary due to the current absence of widely adopted baseline systems or standardised evaluation protocols for open-ended multi-party interactions, where existing solutions are often proprietary, task-specific, or insufficiently documented for direct comparison. Our study addresses the following evaluation questions.

- Q1 How effectively can the system maintain multi-party conversations, particularly in terms of managing turn-taking and recognising participants under different conversational dynamics?
- Q2 What technical barriers, such as latency or recognition errors, most affect interaction quality, and how do these influence user engagement?
- Q3 What aspects of the system’s performance do users identify as needing improvement, and how do these insights align with our quantitative metrics?

## 2 Background and related work

Managing effective conversations involving more than two speakers has long been an important goal. In this section, we present an overview of the main challenges in multi-party conversations, drawing on our experiences and previous work in the field, and highlight the importance of certain cues in conversational agents. Then, we analyse how three recent related studies approached multi-party conversations.

### 2.1 Challenges

Addlesee et al. (2024) recently approached the problem of multi-party conversations. In their work, they mention several challenges that increase the difficulty of this task, namely speaker recognition, turn-taking, group tracking, and goal tracking.

Speaker recognition is the task responsible for identifying the person speaking. This increases the accuracy of the transcribed dialogue – as dialogue is attributed to the correct speaker – which in turn improves the relevance of the generated responses. Speaker recognition can be achieved through voice or face recognition. Through voice recognition, the speech is analysed, and the speaker is identified in a relative or absolute way (Park et al., 2022). Relative voice recognition labels each recorded utterance with a user ID valid within the conversation. However, one person might receive multiple IDs throughout the conversation. Absolute speaker recognition solves this issue by uniquely identifying users across conversations through a prerecorded sample of their voice. Face recognition enables absolute speaker recognition (Adjabi et al., 2020) by extracting features from the face and building a user profile, to which detected faces can be compared in search of a match.

Turn-taking is the second challenge. People perform it effortlessly, while for a conversational agent this task is still challenging (Skantze, 2021). A conversational agent should find occasions to contribute to the conversation in a non-intrusive manner. However, in practice, identifying such locations in the conversation relies on the analysis of many aspects, such as eye gaze, pragmatically complete sentences, prosody, or even breathing. Relying on pauses between sentences has proven to be an unreliable method (Skantze, 2021), as in-turn pauses are often longer than their inter-turn counterparts. Indeed, many turn switches occur through terminal overlap and interruptions, which should be handled appropriately. To overcome these issues, Lala et al. (2017) tracked the likelihood that the system should take the turn and used backchannels when the turn switch was ambiguous, which lent itself well to the attentive listening task chosen for their evaluation.

Next-speaker selection is an important aspect of turn-taking that must be considered. Determining the addressee of a spoken utterance gives insight into whether the robot should respond, and is fundamental for following the conversation and contributing effectively to it. Bohus and Horvitz (2010) empirically demonstrated how a conversational agent could leverage synchronised gaze, gesture, and speech to regulate turn-taking in multi-party settings. Their work introduced a framework that enabled the agent to shape conversational dynamics by using verbal and non-verbal cues, such as directed gaze and eyebrow raises, to influence who would speak next. While this research underscored the potential of multimodal cues not only for managing floor control but also for creating more natural and engaging group interactions, it had the limitation of being based on a task guided by the robot and modelled as a finite state machine, with a specific conversation flow and a predefined set of questions and expected answers.

The challenge of group tracking involves investigating the group dynamics. For instance, detecting whether the group of conversational participants consists of an actual group or separate individuals having different conversations – a situation not uncommon at a busy help desk, to give an example. In this work, we investigate whether an LLM can handle both cases.

The last challenge mentioned is goal tracking. To effectively perform goal tracking, the participants’ goals are determined throughout the interaction to improve the generated responses. This becomes difficult in multi-party conversations as people might have shared goals, different goals, or talk about other people’s goals (Murali et al., 2023).

However, there are many more challenges in handling group interactions. For instance, in their scoping review of computational challenges in social group human-robot interactions, Nigro et al. (2024) highlight engagement detection as another important topic. Indeed, engaging each conversational participant equally is important to facilitate an effective and successful conversation (Murali et al., 2023; Moriya et al., 2012). According to their findings, the most used features to measure engagement are audio features, such as prosody, and video features, such as gaze direction. Furthermore, they report how disengagement often occurs in groups where participants exclude the robot and only continue interacting with each other. Including anthropomorphic characteristics in robots is related to improved engagement (Mehmood et al., 2024; Ghiglino et al., 2021). In this both verbal and non-verbal aspects contribute to this perception (Chidambaram et al., 2012).

The verbal component of improved engagement requires a complete understanding of the interaction and the ability to generate human-like responses (Gibson, 2000). These responses should be natural in their structure and sound in their contents. Furthermore, the latency with which responses are generated must be considered, as long waiting times contribute negatively to the perceived agency (Skantze, 2021).

At the same time, the design and behaviour of the embodied agent will influence the perceived agency. Aspects of turn-taking behaviour, breathing, or other normally unconscious behaviours contribute to this perception. Consequently, it is important to model the robot’s non-verbal aspects consistently (Chidambaram et al., 2012). Inconsistencies and mismatches between these aspects lead to what is called *uncanny valley* (Mori et al., 2012), which entails not only lower perceived agency but often a sense of revulsion.

### 2.2 Related work on multi-party open-ended conversations

Before the advent of LLMs, approaches to multi-party dialogue were grounded in theories developed primarily for two-party interactions, which were then adapted and extended to account for the additional complexity of group settings (Branigan, 2006). Researchers like Clark and Schaefer (1992) proposed that multi-party dialogues are governed by the same foundational principles as two-party ones, though the dynamics are more complex due to the presence of side participants and multiple potential addressees. Among these principles, the collaborative accumulation of common ground and the *Principle of Responsibility* play an important role. These state that each conversation participant is responsible for keeping track of the conversation contents and for allowing other participants to do the same, while at the same time speakers collaborate with addressees directly, and indirectly with side participants to achieve mutual understanding. In practical systems, this theoretical distinction was operationalised by assigning and tracking participant roles (Clark and Carlson, 1982): speaker, addressee, and side-participant. Using explicit heuristics, dialogue tags, or interaction context, alongside simplified interactions and strong dialogue policies, allowed the system to model obligations and expectations for grounding and turn-taking.

The contextual understanding of LLMs overcame many of the technological limitations of previous implementations; as a result, it is not necessary to exactly model the possible conversation contents, as the models will infer the missing information based on the contents of their vast training data. For instance, Addlesee et al. (2023) investigated how LLMs can be used to perform multi-party goal tracking in hospital settings. They introduced a corpus of 29 multi-party conversations between patients, their companions, and a social robot in a memory clinic. The conversations are annotated to track the goals of the participants. They evaluated different models and prompt techniques. Among these, GPT-3.5-turbo, when guided with reasoning-style prompts, significantly outperformed the other models. This kind of goal tracking is fundamental in task-oriented multi-party conversation, while, for conversational-oriented systems, dialogue flow or speaker recognition might be enough to fill in the gaps and provide context-aware robot behaviour.

In a later work on a similar multi-party setting, Addlesee et al. (2024) achieved satisfactory results using the open-source LLM Vicuna for response generation and turn-taking features. Their goal was to provide a system that is practical and entertaining within the context of healthcare clinics. As this LLM runs locally, it avoids network traffic latencies and privacy concerns. The authors, however, did not implement non-verbal features of the robot, which reduces the robot’s capacity to achieve realistic turn-taking during the interaction. They also did not implement speaker recognition, which makes it harder for the conversational AI to produce useful responses toward the specific speaker and track this person throughout the interaction.

Murali et al. (2023) created a meeting facilitation robot for group decision-making. Although this system was not created to handle open-ended conversations, it can handle other aspects of multi-party conversations, such as diarisation and participant engagement. Their findings showed that confidence levels increased in groups that had the robot mediator. The effectiveness also increased as the robot ensured that participants stayed on target. However, the robot did not actively participate in the conversation, reducing the need for accurate turn-taking capabilities.

The evolution of multi-party dialogue systems reflects a shift from rule-based, heuristic-driven approaches to the more flexible, context-aware capabilities offered by modern LLMs. While early systems relied on explicit role assignment and simplified interaction models, recent advancements leverage the inherent contextual understanding of LLMs to address challenges like goal tracking, speaker recognition, and engagement detection. However, gaps remain, particularly in integrating non-verbal cues, managing real-time turn-taking, and achieving fluid conversations. These limitations underscore the need for holistic systems that combine the strengths of LLMs with robust perceptual and behavioural modelling.

## 3 Proposed multi-party interaction system

### 3.1 Requirements

The design and implementation of the multi-party interaction system was guided by the goal of achieving open-ended, multi-party spoken interactions, from which several requirements follow.

The implementation must track the ongoing discourse and match each participant with their contribution. The first requirement is necessary for handling interactions without a specific goal, where the exact words spoken can influence future conversation turns. The second follows from the goal of interacting with multiple speakers, including the possibility of addressing them individually, which requires accurately identifying the author of each statement.

The system must be capable of contributing to the conversation, either through pertinent comments or relevant questions on the topic discussed. To achieve this, the system must understand when it is most appropriate to intervene and when to yield the turn to other participants, while also handling interruptions and barge-ins.

Additionally, the system must display non-verbal cues, such as gaze and eye movement, which improve the handoff to another speaker.

Finally, the implementation must meet three additional requirements. First, minimising delays is critical to ensure responsiveness and maintain the flow of natural conversation. Second, the system must be designed for expandability to accommodate future advancements or additional functionalities. A modular architecture allows for the integration of new capabilities without requiring a complete redesign, ensuring long-term adaptability of our system beyond the scope of its evaluation. Third, the system should remain independent of the embodiment’s hardware to ensure versatility across different platforms and physical forms, as we do not want to be constrained by specific hardware limitations.

### 3.2 Components overview

**FIGURE 1:**
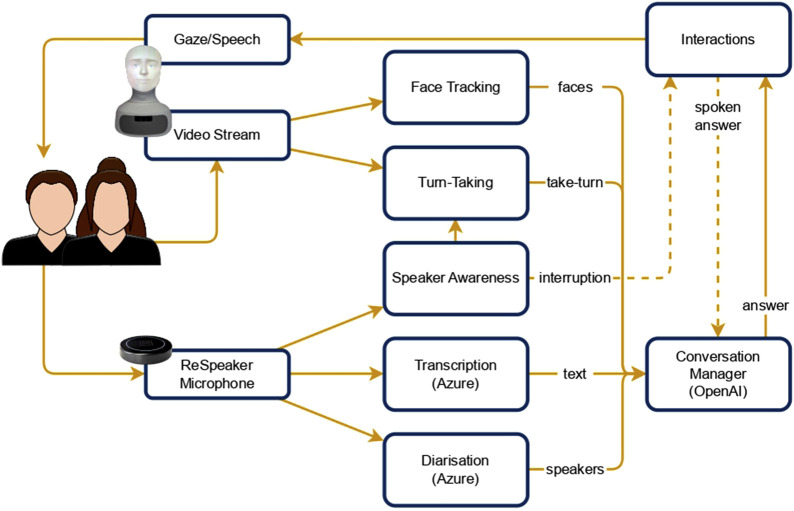
Simplified overview of the information flow between components. The Conversation Manager receives information on the faces and speakers detected, the transcribed text, and whether it should take the turn. Then, it produces an answer and an addressee that are returned to the robot. In case of a user interrupting the robot (dashed lines), the system saves in the conversation only the part of the answer that was actually spoken.

To satisfy the requirements previously introduced, we implemented a modular, event-driven software architecture that allows easy integration of new features and flexibility across different hardware platforms. Each module in the system is designed to handle a specific set of tasks associated with multi-party interactions, such as speaker awareness, verbal and non-verbal interactions, voice recognition, face tracking, speaker diarisation, and turn-taking. A conversation manager module integrates all of these elements to orchestrate the system’s behaviour. A description of each module is provided below, while additional implementation details are reported in the Supplementary Material. Figure 1 shows an example of the information flow between components.

The Speaker Awareness Module uses an external microphone to determine the direction of the speaker’s voice, thereby identifying when conversational turns shift among participants and the robot. A ReSpeaker^1^ microphone array is used to perform this task and enhance the audio capture, which helps the system to accurately identify speakers during multi-party conversations. This module generates events such as detected speaker angles, turn changes, and the start or end of a user or robot’s speech.

The Transcription Module transcribes spoken words in real time using Azure Cognitive Services’ speech-to-text capabilities. This module continuously records and transcribes each utterance and keeps track of the last spoken word to manage the conversational flow. By integrating with the speaker awareness module, the system avoids picking up and transcribing its own speech.

The Interactions Module manages both verbal and non-verbal interactions. It leverages the robot’s – in our case, the Furhat robot – text-to-speech capabilities for verbal responses. For non-verbal cues, it controls the eye gaze and head movements, looking towards the location of the user currently talking. This module can also handle interruptions, pausing the robot’s speech when users begin talking. Although Furhat can display facial expressions, this study focuses primarily on gaze and head movements as interaction tools.

The Diarisation and Face Tracking Modules are responsible for identifying participants through voice and face recognition. Voice recognition uses Azure Cognitive Services to uniquely identify users’ voices across interactions, while face tracking utilises Furhat’s built-in capabilities along with Python’s face recognition library^2^ to recognise users across multiple sessions. This enables continuity in user interactions and allows for personalisation in future engagements, such as user preferences and history of previous interactions. Azure’s services require an initial enrolment phase for each new conversation participant. This phase consists of reading a sentence out loud so that the system can obtain a sample of the user’s voice. The sentence is the same for all users, and the enrolment happens before the actual interaction begins. A similar process is used to enrol faces. The system combines voice and face recognition based on the voice direction of arrival and the face position in the video frame. In the case of conflicts, priority is given to face recognition as it has proven to be more reliable.

The Turn-Taking Module uses data from the other modules, including face orientation and user position, to determine when the robot should take or yield a turn in the conversation. Face orientation cues play a critical role in the system’s decision-making process regarding turn-taking. The system takes the turn if the robot is looked at by the last speaker or after a prolonged silence. The use of silence instead of more sophisticated techniques (Skantze and Irfan, 2025) is justified with the assumption that the users are going to pass the turn through gaze. We plan to experiment with these techniques in the future. In case of failure, when a user starts talking while the robot has initiated its turn, the *Interactions* module pauses the robot’s speech and gives the turn to the user. If the user stops as well, then the robot resumes from where it was stopped.

The Conversation Manager Module acts as the central control point, integrating the outputs of all other modules. Using GPT-3.5 (the latest version available at the time of this study) for response generation, this module ensures timely and contextually relevant contributions, managing the overall flow and content of the conversation. Using the data it receives from the other components, the conversation manager keeps track of the ongoing conversation, where each utterance is associated with its respective author using a best effort approach. In case of failure of the speaker recognition for a specific utterance, the LLM is able to infer the missing information. To select the addressee, the conversation manager provides this conversation history and the list of recognised users from the face tracking module to the LLM, with the instructions to explicitly select an addressee and produce a response. For the exact prompt used, please refer to the Supplementary Material. We used the *stream* option of OpenAI APIs, meaning that the response is immediately available as soon as it is generated. Currently, to generate an answer, the system uses only the transcribed text; in the future, we plan to extend support to images from the camera stream for improved contextual awareness (Abbo and Belpaeme, 2025).

The system is deployed on the Furhat robot (Al Moubayed et al., 2012), a talking head with advanced facial expression capabilities projected onto the robot’s face. Furhat is equipped with a built-in camera for detecting and tracking faces, high-quality speakers for auditory output, and text-to-speech capabilities that allow it to generate expressive and natural-sounding speech. Additionally, Furhat has a customisable face that can be projected to convey different personas and emotions, making it ideal for engaging in social interactions, which is crucial for studying multi-party interactions. The robot allows for flexible control of gaze and head movements, which ensures effective non-verbal communication cues. These features make Furhat a suitable platform to evaluate our system.

## 4 Methodology

### 4.1 Procedure

This study employs a multi-phase design to evaluate the social and conversational performance of the proposed multi-party interaction system deployed on the Furhat robot. The evaluation was designed to capture both technical performance and user perceptions across two distinct interaction settings. Given the absence of established baselines for open-ended multi-party human-robot interaction, our approach focuses on exploratory analysis to assess system capabilities and identify key challenges in this emerging area.

Participants were recruited in pairs to ensure familiarity and promote natural, fluent conversations, as it has been done in previous research (Bohus and Horvitz, 2010). Prior to the experiment, participants provided informed consent in accordance with the guidelines approved by Ghent University’s Ethics Committee. A pre-test questionnaire was used to assess participants’ prior perceptions and knowledge of social robots. The pre-test included items on robot familiarity, general attitude toward technology, and prior exposure to AI systems. A short enrolment step followed, required for Azure’s identification service. Each participant read a short scripted sentence aloud, enabling the system to create a voice profile.

**FIGURE 2:**
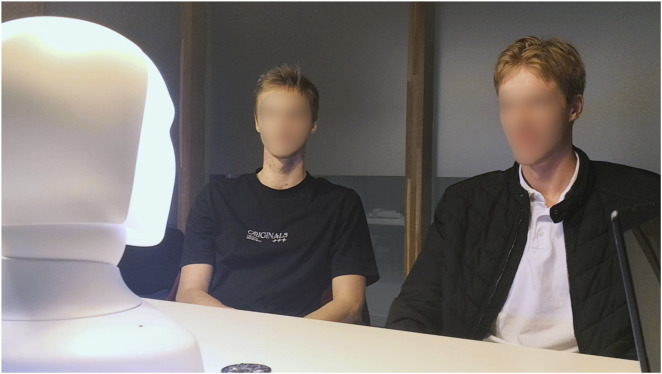
A frame from the camera capturing the *ground truth* showing two subjects interacting with the Furhat robot (on the left); the directional microphone is partially visible at the bottom.

The experiment consisted of two open-ended scenarios. In both, participants sat facing the Furhat robot, as illustrated in Figure 2, and all parties in the conversation spoke English. A directional microphone array and a wide-angle camera were positioned in front of the participants to support the system’s perception modules and to record ground-truth audio-video data for later annotation. The order of the two scenarios was counterbalanced across participant pairs to minimise order effects.

In the parallel scenario, the robot alternated its attention between the two users, responding to each participant in turn. The participants were instructed to each have a conversation with the robot on a topic of their choice, and to switch after a couple of turns with the robot were completed. Although the conversation remained open-ended, this setting encouraged the robot to maintain two loosely independent dialogue threads. The scenario assessed the system’s ability to recognise speakers, track individual conversational goals, maintain coherence with each participant’s conversational direction, and manage alternating turns under overlapping demands. Note that, while turn-taking accuracy was not directly scored, we examined the success of alternating interactions and robot responsiveness through the timing and content of turn shifts. Goal-tracking was evaluated by examining whether Furhat’s responses aligned with the intended conversational topic and the task of each user (e.g., discussing holiday preferences or movie genres), as coded from transcripts and dialogue intents.

The group scenario introduced a more open-ended and natural conversation environment, where both participants and the robot engaged in a shared discussion without prescribed turn-taking rules. Here, participants were told to have an open-ended conversation on a topic of their choice with each other and the robot. In this sense, Furhat actively participated, contributed content, and managed its turns within a fluid, social dialogue. This condition tested the robot’s ability to operate within a single conversational space, dynamically recognise addressees, and contribute coherently to an evolving group dialogue.

No explicit conversational roles were assigned to either the participants or the robot. Furhat was not framed as a moderator, facilitator, or task-oriented agent. Conversations were open-ended, no constraints were imposed on conversation length, and interactions were allowed to end naturally at the participants’ discretion. This design choice was made deliberately to observe how turn-taking, addressee selection, and conversational flow emerged in a multi-party human-robot interaction setting without being shaped by predefined roles or scripted interaction patterns.

After each scenario, participants completed three standardised questionnaires: the Robotic Social Attributes Scale (RoSAS) (Carpinella et al., 2017), which measures perceived friendliness, competence, and animacy; the Interaction Quality scale (IQ) (Schmitt and Ultes, 2015), which assesses fluency, satisfaction, and responsiveness; and the Multidimensional Measure of Trust (MDMT) (Malle and Ullman, 2021), which evaluates participants’ trust in the robot. Each has been applied across various contexts in human-robot interaction literature, including robot acceptance, social engagement, and user satisfaction (Roesler et al., 2022). Questionnaires were administered separately for each scenario to obtain scenario-specific perceptions. Responses included 5-point Likert scales and optional open-ended comments, inviting participants to elaborate on positive impressions, difficulties, and desired improvements. Full questionnaire items are listed in the Supplementary Material.

Social presence was assessed through a binary question (*“Did you feel a sense of social presence from Furhat during the conversation?”*) and is reported separately in Table 1, as it represents a qualitative threshold (presence vs. absence) rather than a graduated experience. This binary measure was not included in the User Experience construct mean, which comprises only Likert-scale questions assessing conversation naturalness and interaction enjoyment. This approach aligns with standard practice in HRI research where social presence is treated as a categorical outcome Carpinella et al. (2017).

**TABLE 1:** Example of survey responses regarding engagement and performance.

Construct	Question	Settings	Yes (%)	No (%)
Engagement	Did you feel a sense of social presence from Furhat during the conversation?	Group	66.7	33.3
		Parallel	50.0	50.0
Performance	Did Furhat interrupt or speak over you or your partner?	Group	60.0	40.0
		Parallel	56.7	43.3
Performance	Did Furhat consistently identify the correct speaker (you or your partner) when responding to questions or comments?	Group	73.3	26.7
		Parallel	66.7	33.3

Each evaluation question is operationalised through specific metrics: Q1 (conversation management) is assessed via addressee detection accuracy (Table 2) and the RoSAS competence subscale (Table 1); Q2 (technical barriers) via recognition accuracy metrics (Table 3) and IQ responsiveness items; Q3 (user-identified improvements) via qualitative open-ended responses analysed thematically (Section 5.3).

**TABLE 2:** Addressee detection accuracy (percentages); *blank* denotes instances with no addressee detected or recognised.

Type	Correct	Incorrect	Blank
Addr. detection (group)	79.3 (3.8 interest.)	13.6	7.2
Addr. detection (parallel)	92.6 (1.7 interest.)	7.0	0.4

**TABLE 3:** Voice and face recognition accuracy (percentages).

Type	Correct	Incorrect	Blank
Voice recognition (group)	18.4	4.5	77.0
Voice recognition (parallel)	26.8	0.4	72.8
Face recognition (group)	80.0	19.5	0.5
Face recognition (parallel)	94.7	4.2	1.1

*Correct recognition* was defined as a match between the system’s prediction and the speaker’s identity as verified through annotated video ground truth; *incorrect recognition* reflected mismatches, while *blank* denotes instances with no speaker ID detected or recognised. Here, *voice recognition* refers to real-time speaker identification (matching utterances to known speaker profiles), not diarisation or speech-to-text transcription.

### 4.2 Evaluation metrics

Alongside the subjective questionnaires, we collected system-level performance metrics to objectively assess the system’s effectiveness of the multi-party interaction system. All conversation logs, system outputs, and audio-video recordings were synchronised and manually annotated to support accurate scoring.

Conversation length. Each scenario lasted on average 12.4 min (SD=3.1) . The number of conversational turns and the distribution of speech across participants were extracted from transcripts to quantify engagement and participants’ balance.

Speaker recognition accuracy. Speaker identity was estimated using two modalities: (i) Azure’s speaker identification API for audio-based recognition, and (ii) Python’s face recognition library for visual recognition. For both modalities, the predictions were aligned with manually annotated ground-truth labels to determine correctness.

Addressee detection accuracy. For every robot utterance, annotators labelled whether Furhat directed its response to the correct user based on the preceding conversational context and ground-truth recordings. Addressee labels were classified as: *correct* response directed to the intended speaker, *incorrect* response directed to the wrong speaker, or *inclusive* the robot intentionally addressed both users within a shared response, despite potential limitations in non-verbal alignment.

Latency. It was defined as the time elapsed from the end of the user’s utterance transcription to the onset of the robot’s verbal response. This includes multimodal processing, intent inference, and LLM generation time. Transcription delay was excluded from this measure, as it depends on the external Azure speech recognition service. The LLM was run in streaming mode to reduce response delay.

Goal-tracking accuracy. Dialogue transcripts were annotated to determine whether the robot’s responses were coherent with the user’s conversational goal at each turn. Coders assessed topic consistency, response appropriateness and the extent to which the robot maintained or shifted the ongoing conversational direction.

All annotations were performed by one author, and independently reviewed by a second author for speaker identification, addressee detection, and goal-tracking accuracy. Inter-rater reliability was assessed using Cohen’s κ ( κ=0.83 for addressee detection, κ=0.79 for goal-tracking). Disagreements were resolved through discussion with a third author.

This combination of quantitative system metrics and subjective user evaluations provides a comprehensive perspective on the robot’s multi-party conversational performance across both scenarios.

### 4.3 Participants

Thirty young adults participated in the study (8 female, 22 male; $M_{\text{age}}=22.7$ , SD=1.88 ). Participants were recruited in familiar pairs to encourage natural, comfortable interaction and to reduce social inhibition during multi-party dialogue. Participants were recruited through snowball sampling via personal and friends-of-friends networks. No compensation was provided.

Most participants (73.3%) reported no prior experience with social robots, while 20.0% had minimal exposure, and only 6.7% had previously interacted with a social robot. All participants were fluent English speakers and reported no hearing or speech impairments. Participation was voluntary, and all procedures were approved by the Ethics Committee of Ghent University. Data were collected anonymously, and participants were free to withdraw at any time.

## 5 Results and discussion

To evaluate the performance and perceived quality of our conversational system, we analysed quantitative metrics, participant survey responses, and qualitative feedback collected across both interaction settings. The following subsections synthesise these findings to address our three evaluation questions: how well the system manages multi-party dialogue (Q1), what technical barriers hinder performance (Q2), and which aspects users identify for improvement (Q3). Each subsection highlights distinct but interrelated dimensions of interaction quality, enabling a comprehensive discussion of system strengths, limitations, and opportunities for future refinement.

### 5.1 Conversation management performance

To answer Q1 on how effectively the system managed multi-party interaction, we examined turn-taking behaviour, addressee detection, and conversational coherence across both interaction settings.

User Experience ratings (conversation naturalness and enjoyment) were higher in group settings ( M=3.40 , SD=0.47 ) compared to parallel settings ( M=3.23 , SD=0.47 ), though both conditions showed similar variability across questions. As illustrated in Tables 4–8, group interactions also showed a higher concentration of positive ratings across several engagement- and experience-related constructs, suggesting that shared conversational spaces fostered stronger perceptions of involvement and social presence.

**TABLE 4:** Engagement construct - rating distribution.

Question	Cond	1	2	3	4	5	M	SD
Q1	G	0 (0.0)	1 (3.3)	9 (30.0)	17 (56.7)	3 (10.0)	3.73	0.69
	P	0 (0.0)	1 (3.3)	8 (26.7)	19 (63.3)	2 (6.7)	3.73	0.64
Q2	G	0 (0.0)	3 (10.0)	13 (43.3)	9 (30.0)	5 (16.7)	3.53	0.90
	P	0 (0.0)	8 (26.7)	6 (20.0)	13 (43.3)	3 (10.0)	3.37	1.00
Q3	G	0 (0.0)	3 (10.0)	9 (30.0)	15 (50.0)	3 (10.0)	3.60	0.81
	P	1 (3.3)	4 (13.3)	10 (33.3)	14 (46.7)	1 (3.3)	3.33	0.88
Q4	G	0 (0.0)	2 (6.7)	6 (20.0)	13 (43.3)	9 (30.0)	3.97	0.89
	P	0 (0.0)	3 (10.0)	4 (13.3)	14 (46.7)	9 (30.0)	3.97	0.93

N = 30 per condition. Values show n (%). Ratings: 1 = lowest, 5 = highest. G = Group; P=Parallel; = same question as above.

Questions:

Q1: Engagement level.

Q2: Interest in contributions.

Q3: Personality/demeanor fit.

Q4: Motivation to participate.

**TABLE 5:** User experience construct - rating distributions.

Question	Cond	1	2	3	4	5	M	SD
Q1	G	1 (3.3)	5 (16.7)	16 (53.3)	7 (23.3)	1 (3.3)	3.07	0.83
	P	0 (0.0)	10 (33.3)	13 (43.3)	7 (23.3)	0 (0.0)	2.90	0.76
Q2	G	0 (0.0)	2 (6.7)	8 (26.7)	16 (53.3)	4 (13.3)	3.73	0.78
	P	0 (0.0)	2 (6.7)	12 (40.0)	13 (43.3)	3 (10.0)	3.57	0.77

N = 30 per condition. Values show n (%). Ratings: 1 = lowest, 5 = highest. G = Group; P=Parallel; = same question as above.

Questions:

Q1: Naturalness of conversation flow.

Q2: Interaction enjoyment.

**TABLE 6:** Usability construct - rating distributions.

Question	Cond	1	2	3	4	5	M	SD
Q1	G	0 (0.0)	1 (3.3)	7 (23.3)	16 (53.3)	6 (20.0)	3.90	0.76
	P	0 (0.0)	4 (13.3)	8 (26.7)	12 (40.0)	6 (20.0)	3.67	0.96
Q2	G	0 (0.0)	1 (3.3)	8 (26.7)	16 (53.3)	5 (16.7)	3.83	0.75
	P	0 (0.0)	5 (16.7)	6 (20.0)	16 (53.3)	3 (10.0)	3.57	0.90

N = 30 per condition. Values show n (%). Ratings: 1 = lowest, 5 = highest. G = Group; P=Parallel; = same question as above.

Questions:

Q1: Clarity of visual cues.

Q2: Ease of interaction.

**TABLE 7:** Performance construct - rating distributions.

Question	Cond	1	2	3	4	5	M	SD
Q1	G	0 (0.0)	0 (0.0)	6 (20.0)	15 (50.0)	9 (30.0)	4.10	0.71
	P	1 (3.3)	5 (16.7)	3 (10.0)	10 (33.3)	11 (36.7)	3.83	1.21
Q2	G	0 (0.0)	2 (6.7)	5 (16.7)	10 (33.3)	13 (43.3)	4.13	0.94
	P	1 (3.3)	3 (10.0)	3 (10.0)	16 (53.3)	7 (23.3)	3.83	1.02
Q3	G	1 (3.3)	2 (6.7)	7 (23.3)	12 (40.0)	8 (26.7)	3.80	1.03
	P	1 (3.3)	0 (0.0)	2 (6.7)	15 (50.0)	12 (40.0)	4.23	0.86

N = 30 per condition. Values show n (%). Ratings: 1 = lowest, 5 = highest. G = Group; P=Parallel; = same question as above.

Questions:

Q1: Understanding of questions.

Q2: Relevance of responses.

Q3: Accuracy of information.

**TABLE 8:** Turn-taking construct - rating distributions.

Question	Cond	1	2	3	4	5	M	SD
Q1	G	0 (0.0)	4 (13.3)	8 (26.7)	12 (40.0)	6 (20.0)	3.67	0.96
	P	0 (0.0)	4 (13.3)	8 (26.7)	14 (46.7)	4 (13.3)	3.60	0.89
Q2	G	0 (0.0)	3 (10.0)	10 (33.3)	13 (43.3)	4 (13.3)	3.60	0.86
	P	0 (0.0)	1 (3.3)	7 (23.3)	15 (50.0)	7 (23.3)	3.93	0.78

N = 30 per condition. Values show n (%). Ratings: 1 = lowest, 5 = highest. G = Group; P=Parallel; = same question as above.

Questions:

Q1: Intuitiveness of turn-taking.

Q2: Appropriateness of timing.

Turn-taking dynamics differed noticeably between conditions. In the parallel scenario, conversational alternation between users remained relatively stable, and participants found it easier to anticipate when the robot would respond. In the group condition, however, the conversational flow became more fluid and spontaneous, leading to greater uncertainty about turn location. As shown in Table 1, interruptions or overlapping speech occurred in 60.0% of group turns compared with 56.7% in the parallel settings. Participants often expressed uncertainty during specific moments of the interaction about their conversational turns or the intended recipient of the robot’s responses, particularly during overlapping speech. This uncertainty reflects transient interactional ambiguity rather than participants’ overall retrospective assessment of the robot’s speaker identification performance.

Addressee detection accuracy aligned with these observations. The system correctly directed its responses in 92.6% of cases in the parallel condition but only in 79.3% of cases in the group condition (see Table 2). Participants reported that when the robot’s gaze direction, head movements, and verbal cues aligned, turn-taking felt intuitive and easy to follow. One participant noted that *“the robot’s eye movements and head-turning made it clear when it was my turn to speak.”* However, when verbal output conflicted with nonverbal behaviour, participants experienced brief ambiguity, particularly during rapid turn-switching or overlapping utterances.

Despite these inaccuracies, participants reported a stronger sense of social presence during the group setting (66.7%) than during the parallel setting (50.0%) , as shown in Table 1. This suggests that participants’ global retrospective evaluations can remain positive even when brief ambiguities occur during real-time multi-party interaction, as the robot’s attempts to engage both users through inclusive addressing contributed to a more socially engaging atmosphere.

Three dialogue excerpts illustrate the strengths and limitations of the system’s turn management.The following transcript excerpts are presented for illustrative purposes to highlight characteristic system behaviours. Overlaps and interruptions are indicated explicitly in the text to clarify interaction dynamics.

The first highlights misaddressing in the presence of an interruption:

**User A:** “I’ve gotten into cycling recently, especially long weekend rides.”

**Robot (addressing User A):** “That sounds amazing! Do you ride solo or with a group?”

**User B (overlapping the robot’s question to User A)**: “Have you tried mountain biking?”

**Robot (misaddressing User A):** “Mountain biking adds a whole new challenge. What trails do you usually ride, User A^3^?”

Here, the system maintained the original conversational thread with User A, which preserved coherence but failed to acknowledge User B’s overlapping contribution. This behaviour highlights the trade-off the system must make between stability and responsiveness in multi-party scenarios.

The second example demonstrates moments of inclusive addressing, where the LLM extends the scope of the response to engage both participants:

**Robot (addressing User A):** “That’s awesome, User A! What do you enjoy most about roller coasters?”

**User A: “I really like the ones that go really fast.”**

**Robot (addressing User A): “Speedy roller coasters do give a thrilling rush! User B, are you also up for the fast-paced rides?”**

Participants often interpreted such turns positively when accompanied by corresponding gaze cues, viewing them as socially inclusive. However, when nonverbal cues did not shift accordingly, the inclusivity occasionally produced ambiguity about the intended addressee.

The third example shows how the system reused contextual information across participants:

**User A:** “Do you want to talk about South America, for example, Peru? Because I’m going there in like 2 weeks, maybe.”

**Robot (addressing User A):** “Peru sounds amazing! Any particular places in Peru you’re excited to visit?”

Conversation continues…

**User B:** “Hey, I’d love to discuss some travel destinations. What’s the first destination that comes to your mind?”

**Robot (addressing User B):** “Peru comes to mind as a fascinating travel destination with its rich history and diverse landscapes. Have you ever considered visiting South America?”

Here, the system later responded to User B by drawing on relevant contextual information. This memory context helped maintain topic continuity and was perceived as coherent when it aligned with the ongoing conversational flow.

Participants in parallel sessions particularly appreciated the clarity afforded by higher addressee accuracy and simpler conversational dynamics. Personalised addressing – such as using names or maintaining prolonged eye contact – was frequently mentioned as enhancing engagement. In contrast, the additional ambiguity present in group interactions sometimes weakened these positive effects, underscoring the need for improved multimodal integration and more reliable addressee identification.

Underlying these behaviours is the system’s LLM-driven addressee-selection logic, which typically prioritised the most recent speaker to maintain conversational flow. When this logic aligned with gaze and head movements, the coders noted smooth transitions and coherent turn allocation. However, in cases where verbal reasoning diverged from nonverbal cues or where overlapping speech created inherent ambiguity, the system occasionally broadened its responses or misassigned turns. While inclusive addressing could be socially engaging, the absence of supporting nonverbal shifts sometimes introduced uncertainty about the intended recipient.

Taken together, these findings indicate that the system can sustain coherent multi-party interaction and demonstrates emerging strengths in inclusive engagement and topic continuity, particularly in structured parallel settings. However, precise turn-taking and reliable addressee identification remain challenging in more dynamic, overlapping conversational contexts. Improving multimodal signal integration, refining addressee detection logic, and developing more responsive turn-taking mechanisms will be essential for advancing multi-party conversational capabilities in social robots.

### 5.2 Technical barriers

Multi-party social interaction places substantial demands on real-time perception and response generation. Across both conditions, several technical behaviours consistently shaped the naturalness and fluidity of the interaction. Addressing Q2, three factors emerged as the most influential: (i) the difficulty of audio-based speaker identification during overlapping or rapid turn-taking, (ii) inconsistencies in multimodal cue integration, and (iii) response latency.

Audio-based speaker recognition emerged as a notably problematic component across both interaction settings, although its limitations were more pronounced during group interaction. As shown in Table 3, only 18.4% of group utterances and 26.8% of parallel utterances were correctly attributed, with most remaining instances categorised as unrecognised. These low percentages do not indicate malfunction but reflect the inherent difficulty of recognising speakers in multi-party environments characterised by short interjections, overlapping speech, and rapid turn transitions. Participants frequently attributed breakdowns in interaction to these audio recognition limitations, with comments such as: *“I had to repeat myself several times because it didn’t know who was talking.”* These observations reinforce that improving robustness under conditions of crosstalk and simultaneous contributions is essential for natural multi-party dialogue.

In contrast to audio, visual speaker detection performed reliably across conditions, achieving 80.0% accuracy in group interactions and 94.7% accuracy in parallel interactions. Annotators pointed out that when voice recognition failed, visual identification often enabled the robot to maintain coherence in its response. However, when audio and visual predictions diverged, the system occasionally produced misaligned behaviours, such as looking at one participant while replying to the other. As one participant noted: *“At times, Furhat clearly looked at me but responded to my partner, causing confusion about whom it was addressing.”* These mismatches highlight the need for confidence-weighted multimodal fusion that can dynamically prioritise the more reliable modality – typically vision – during periods of overlapping speech or rapid turn-switching.

Latency constituted the second major barrier to fluid interaction. The average response delay was 1.35 s (SD = 0.55), occasionally reaching nearly 5 s. These delays disrupted group dialogues where participants often shifted topics or resumed speaking before the robot responded. Participants reported that the robot occasionally felt slow or detached: *“The delay made the conversation feel sluggish,”* and *“It broke the rhythm.”* Analysis of system logs indicates that LLM generation time was the largest contributor to this delay (mean 0.76 s). This suggests that future iterations would benefit from lightweight or predictive generation strategies, particularly for fast-paced social interactions.

**FIGURE 3:**
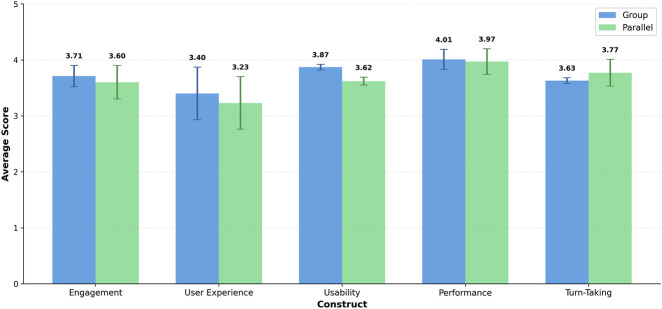
Average ratings for each construct across Group and Parallel conditions. Error bars represent standard deviation across questions within each construct, indicating variability in responses. Social presence, being a binary measure, is reported separately in Table 1. Each construct has two bars (Group in blue, Parallel in green) allowing direct comparison between conditions.

These technical behaviours were directly reflected in post-interaction questionnaire responses (Table 1). Participants reported positive engagement in both scenarios, with group interactions slightly outperforming parallel ones for engagement (M = 3.71, SD = 0.07 vs. M = 3.60, SD = 0.13), as shown in Figure 3. Similarly, perceptions of social presence were higher in the group condition (66.7%) than in the parallel condition (50.0%).

Qualitative responses support these findings. Participants frequently described the group scenario as more lively, socially dynamic, and conducive to shared engagement, highlighting the robot’s participation in a shared conversational space; whereas the parallel setting was perceived as more structured and predictable.

At the same time, reports of being interrupted were slightly more frequent in the group condition (60.0%) than in the parallel condition (56.7%). This reflects the more dynamic conversational environment of open group dialogue, where overlapping speech and rapid conversational shifts were more likely to occur. Participants in these sessions ocassionally experienced reduced conversational naturalness, increased conversational overlap, and disrupted turn-taking cues. In contrast, the parallel condition, characterised by clearer conversational structure and fewer simultaneous contributions, mitigated some of the negative impact of latency and allowed participants to better accommodate delayed robot responses.

Across both settings, audio recognition fragility, modality conflicts, and LLM-induced delays emerged as the main limiting factors of multi-party performance. These findings highlight the importance of developing more adaptive multimodal fusion strategies, strengthening speaker tracking under overlapping speech, and reducing response latency to support natural, real-time social interaction. Addressing these challenges will be crucial for enabling LLM-driven robots to operate effectively in multi-party settings.

### 5.3 User perceptions and design insights

Participants’ reflections offered a complementary perspective to the objective performance metrics, revealing how the system’s behaviours were interpreted, tolerated, or valued during interaction. While overall engagement ratings were positive across both conditions, open-ended comments indicated specific areas where the interaction could be made more natural, personal, and intuitive.

Across both interaction settings, participants consistently expressed a preference for greater personalisation and contextual awareness in the robot’s behaviour. Although mean ratings for social perception remained above 3.5 in both scenarios, open-ended feedback revealed that several responses felt generic or insufficiently tailored to the individual. Around 16.7% of participants explicitly stated that the robot did not make use of information they had previously shared. One participant remarked, *“It would have been nice if Furhat remembered something I said earlier, bringing it back later to personalise the interaction.”*

The system already included a memory mechanism capable of storing user information, yet the language model did not consistently surface this knowledge in its responses. The participants, therefore, perceived the robot as lacking memory despite the underlying capability being present. This gap illustrates a wider design challenge in LLM-driven systems. Memory must not only exist at the architectural level but must also be expressed in ways that are perceptible and meaningful to users, such as through timely references to earlier statements or personalised conversational follow-ups.

Participants frequently commented on the formal tone of the robot’s utterances. Although 76.7% rated its conversational appropriateness as natural (an overview of the participants’ rating of the robot’s conversational skills is provided in Table 9), many noted that its replies, while accurate, often felt too polished or scripted. Descriptions such as too perfect or overly formal appeared repeatedly in feedback. Participants explained that these characteristics reduced the spontaneity of the interaction and added to the impression of conversational rigidity. This experience was often intensified by response delays, which made the robot feel slow or detached and diminished the perceived naturalness of turn-taking.

**TABLE 9:** Summary of responses from participants on Furhat’s conversational skills.

Question	Yes (%)	No (%)
Did Furhat successfully follow the flow of the conversation between you and your partner?	96.67	3.33
Did Furhat contribute to the conversation in a natural way?	76.67	23.33
Did Furhat effectively provide prompts that encouraged further discussion?	70.00	30.00
In individual conversations, did Furhat differentiate effectively between the speaker and the partner?	86.67	13.33
Did Furhat appropriately respond to personal preferences in the separate conversation?	83.33	16.67
Did Furhat mistakenly involve the other person in your conversation or direct a response incorrectly?	33.33	66.67
Was Furhat’s response length appropriate to allow for a balanced flow of the conversation?	90.00	10.00

Turn-taking cues were another recurring theme in participant reflections. While quantitative results indicated moderate success in identifying speakers, many participants reported difficulty in knowing when to speak or when a response was intended for them. This uncertainty was especially common in the group setting, where conversational dynamics were more fluid. Participants recommended more explicit nonverbal cues, such as clearer gaze shifts, more distinct head turns, or small gestures to signal turn boundaries. Comments emphasised that even subtle adjustments in these behaviours would greatly improve conversational clarity.

User reflections on recognition issues closely paralleled the quantitative findings. Participants frequently noted moments when they felt unheard or misidentified, often linking these incidents to reduced engagement or conversational flow. Statements like *“Sometimes Furhat didn’t respond because it couldn’t hear me clearly,”* or *“I had to repeat myself several times,”* reflect how recognition errors manifest subjectively as conversational breakdowns. Interestingly, users did not typically distinguish between audio and visual recognition; instead, mismatches such as the robot looking at one person while addressing another were experienced simply as inconsistency, reinforcing the importance of more transparent and conflict-resolving multimodal fusion.

Alongside these recommendations, several participants emphasised aspects of the system they found particularly effective. The robot’s nonverbal behaviour, especially its eye contact, head orientation, and subtle facial expressions, was frequently described as engaging and lifelike. Participants also noted that the robot maintained conversational coherence even when recognition errors occurred, which they attributed to its consistent topic-following and contextual reasoning. These observations suggest that users perceive the system as socially capable and expressive, even when technical limitations affect timing or addressee accuracy.

Taken together, these findings provide a response to Q3. Users identified several areas where system behaviour could be improved, most notably in personalisation, spontaneity of language, clarity of turn-taking cues, consistency across modalities, and response timing. At the same time, they recognised strengths in nonverbal expressiveness and overall social presence. These insights outline concrete design priorities for future iterations of multi-party conversational robots.

### 5.4 Limitations and future work

While the study provides clear insights into the capabilities and challenges of LLM-driven multi-party interaction, several limitations should be acknowledged. First, the evaluation involved a modest sample size of 30 young adults, which constrains the generalisability of the findings and does not capture the broader range of conversational styles found in real-world settings.

Second, the interactions were conducted under controlled laboratory conditions. Although the group scenario introduced natural overlap and spontaneity, the structured environment may not fully reflect the complexity and unpredictability of everyday multi-party encounters.

Third, several performance outcomes are closely tied to the specific architectural choices made (e.g., relying on Azure’s speaker identification, GPT-3.5 for response generation). Different recognition pipelines or more recent LLMs may yield substantially different results. Similarly, the relatively short duration of the interactions limits conclusions about long-term adaptation, multi-session memory use, or behaviour over extended deployments. Future research should therefore explore larger group settings, more diverse user populations, and longer-term engagements to assess how LLM-driven conversational architectures scale and adapt beyond controlled experimental setups. This is especially relevant concerning the latencies of the system.

In this work, we took advantage of the streaming feature of the LLM, generating the speech as soon as a complete sentence was produced without waiting for the full output to be completed. However, we did not use incremental speech recognition due to the additional difficulties of the multi-party setting, combined with the modular implementation. In future work, we plan to start generating a response as soon as a certain amount of the transcribed user speech is available. This response can then be ignored or extended when the robot takes the turn, producing a faster response.

## 6 Conclusion

This work presented and evaluated a multimodal conversational architecture enabling a social robot to engage in open-ended dialogue with two users simultaneously. Through a controlled two-condition study, we examined how the system coordinated turn-taking, identified speakers, selected addressees, and responded to rapidly shifting conversational dynamics.

Our findings show that the system can sustain coherent multi-party interaction, particularly in structured parallel settings where addressee accuracy and topic continuity remained high. In more fluid group interactions, the robot demonstrated emerging strengths, such as inclusive addressing and robust visual recognition, while also revealing the challenges posed by overlapping speech, modality conflicts, and response latency. User reflections highlighted both the value of the robot’s social expressiveness and the need for clearer turn-taking cues, stronger personalisation, and more reliable multimodal grounding.

Overall, the study provides a detailed view of the capabilities and limitations of current LLM-driven conversational systems in multi-party contexts. The findings point to several technical priorities for future work, including more adaptive multimodal fusion, improved handling of overlapping speech, and faster response generation to support smoother conversational flow. Extending the system to larger groups, longer-term interactions, and newer language and perception models will help advance the development of socially capable robots that can operate effectively in natural, dynamic, real-world settings.

## Data Availability

The original contributions presented in the study are included in the article/Supplementary Material, further inquiries can be directed to the corresponding authors.

## Footnotes

1
https://wiki.seeedstudio.com/ReSpeaker-USB-Mic-Array/. We observed that using the manufacturer’s microphone case makes its direction of arrival data completely inaccurate, and as such, removed the casing for our study

2
https://github.com/ageitgey/face_recognition

3 The system used the actual name as registered during the enrolment phase
