# Supplementary material for: Multi-party open-ended conversation with a social robot
Source: Front Robot AI. 2026 Apr 15;13:1766383. doi: 10.3389/frobt.2026.1766383 (PMC13124475; doi:10.3389/frobt.2026.1766383)
Supplement: Supplementary file 1 [file Supplementaryfile1.pdf]

Supplementary Material

1 IMPLEMENTATION DETAILS

1.1 Architecture

In the following section, we provide additional details on the architecture and implementation of the system. For each module, we specify its function, what the component publishes and to what it subscribes. Finally, when applicable, we provide a reflection with the assumptions for this component, its limitations, and future works.

1.1.1 Speaker Awareness Module

1.1.1.1 Function.

This module detects the direction of arrival of the user's voice. Using this information, it detects whether there might have been a turn switch between two users or between a user and the robot. For the direction of arrival, the degrees are calculated from the perspective of the robot, having 0 degrees at the right, increasing counter-clockwise. Speech coming from between 180 and 360 degrees is interpreted as coming from the robot. The microphone used is a ReSpeaker USB Mic Array[1] positioned in front of the robot. This device has four microphones and can run speech algorithms on-chip. It is controlled through a Python script following the documentation of the manufacturer.

1.1.1.2 Pub: user-angle.

Every 100 ms, this publishes an integer with the direction of arrival of the audio, unless it comes from the robot's direction.

1.1.1.3 Pub: user-user-switch.

Every 100 ms, this checks whether the direction of arrival has changed by more than 20 degrees with respect to the previous measurement. If so, an event is fired. The measurement ignores audio coming from the robot's direction.

1.1.1.4 Pub: robot-user-switch.

Every 100 ms, fire an event if the direction of arrival has changed from the user to the robot or vice versa. The event details whether the turn switched to or from the robot.

1.1.1.5 Reflection.

This component is implemented with two assumptions: first, that no one is standing behind or on the side of the robot. Second, the participants have at least a 20-degree angle between them and the microphone. Further testing is needed to assess the system's behaviour when these requirements are not met, as in principle it can rely on information from other components to overcome these limitations. The turn-switch logic should be moved to another component, as it can benefit from input from other components. Detecting the robot's voice might take advantage of information from the Interactions Module. The ReSpeaker hardware can run small algorithms to improve the signal and provide additional information, which could improve the system's performance.

[1] https://wiki.seeedstudio.com/ReSpeaker-USB-Mic-Array/

1.1.2 Transcription Module

1.1.2.1 Function.

Transcribes text and identifies participants through the conversation transcriber from Azure Cognitive speech services. The ID assigned to the voices is not consistent across interactions, it only differentiates between the voices detected.

1.1.2.2 Pub: transcribed.

When a result from the transcriber is received, this posts the transcribed text, its start time, and the ID of the speaker.

1.1.2.3 Sub: Speaker Awareness – robot-user-switch.

Pause the transcription when the turn is switching to the robot; resume it when the turn is switching to a user.

1.1.2.4 Reflection.

In the future, we plan to experiment with different speech-to-text engines, including Whisper models, which showed promising performance and support multiple languages.

1.1.3 Interactions Module

1.1.3.1 Function.

This component interfaces with the Furhat robot through the Python remote API provided by the manufacturer. It is used for speaking utterances and controlling the gaze direction. It handles interruptions, as it stops the robot's speech when the users start talking. Finally, it controls the robot's LEDs, which are turned on when the robot is listening.

1.1.3.2 Pub: spoken-text.

Publishes the sentence that has been spoken by the robot.

1.1.3.3 Sub: Speaker Awareness – robot-user-switch.

If the user starts talking while the robot is talking, the robot stops. If no speech from the user is detected for at least 1.5 seconds, the robot continues talking from where it stopped.

1.1.3.4 Sub: Transcription – transcribed.

The robot shows a feedback gesture – *brow raise* – in response to a transcribe event.

1.1.3.5 Sub: Conversation Manager – text.

When this component receives a sentence that the robot should say, the robot's LEDs are turned off. Then, this component sends the received text to the robot's text-to-speech module. The text is not sent if an interruption happened less than two seconds before, or if a person was talking (i.e., a transcription was received) less than one second before. Finally, the LEDs are turned back on to signal that the robot is listening again.

1.1.3.6 Sub: Face Tracking – face-position.

When a user is talking and their face's position is known, this instructs the robot to look at the direction specified.

1.1.3.7 Sub: Face Tracking – face-id.

Instructs the robot to look at the user with the specified ID. The ID is assigned and handled by the Furhat robot.

1.1.3.8 Reflection.

The logic detecting interruptions should be moved outside of this component. In addition, more facial expressions could be included as feedback to the user.

1.1.4 Diarisation Module

1.1.4.1 Function.

This component records short pieces of the conversation and uses Azure speaker recognition's text-independent identification service to assign a unique ID to the voices being recorded. This is slower than the transcription but provides a unique ID. Before the interaction, users are enrolled into the system, by asking them to read out loud a short paragraph.

1.1.4.2 Pub: speaker.

Publish an event when new text is associated with a user.

1.1.4.3 Sub: Transcription – transcribed.

When the text of a transcription is received, this component associates it with the user that has been identified by the Azure services. Then, the audio recording is stopped if it is running.

1.1.4.4 Sub: Speaker Awareness – robot-user-switch.

When the turn switches from the robot to a user, this starts recording the audio to be sent to the speaker identification services, if not already recording. The recording lasts for three seconds, and this is usually enough to identify the speaker.

1.1.4.5 Sub: Speaker Awareness – user-user-switch.

When the turn switches between users, the audio recording is stopped if it is running. The results of the interrupted recognition task, if any, are handled in the background. In the meantime, the recording is restarted to identify the new user.

1.1.4.6 Reflection.

This component assumes that conversation participants have been previously enrolled. The functionality of merging user information under a unique ID should be moved outside of this component.

1.1.5 Face Tracking Module

1.1.5.1 Function.

This component uses the robot's built-in camera and Python's face recognition library[2] to recognise previously enrolled users. The ID assigned to the users is consistent across interactions: the same user will be assigned to the same ID every time. The robot's camera is accessed through the video stream provided by the manufacturer.

[2] https://github.com/ageitgey/face_recognition

1.1.5.2 Pub: users.

Every two seconds, this component takes an image from the robot's video stream, which includes annotations. The location of the annotation's bounding boxes around the detected faces is updated for consistency with the angles detected by the Speaker Awareness module. The image is sent to Python's face recognition library to detect face encodings and compared with the enrolled users. For each recognised user, this component publishes the updated location of the face, the ID assigned by the robot and the confidence of the face recognition.

1.1.5.3 Pub: face-id.

Every time the position of the user currently talking is updated, the ID (assigned by the Furhat robot) of the user currently talking is published.

1.1.5.4 Pub: face-position.

Every time the position of the user currently talking is updated, the angle of the user currently talking is published.

1.1.5.5 Sub: Speaker Awareness – user-user-switch, robot-user-switch.

When a user-user or robot-user turn switch happens, this component uses the stored data about the faces' position and the voices' direction of arrival to estimate which user is talking.

1.1.5.6 Sub: Speaker Awareness – user-angle.

Records the last detected voice direction of arrival. This function ignores variations less than 30 degrees. Contrary to the previous case, this does not assume that a turn switch occurred.

1.1.5.7 Sub: Transcription – transcribed.

When a new transcription is available, this method associates it with the user who is currently talking.

1.1.5.8 Sub: Conversation Manager – addressee.

If the addressee's position is known, it is used to set the current user location.

1.1.5.9 Reflection.

This component assumes that the conversation participants have been previously enrolled. In the future the functionality of merging user information using their face and voice direction should be moved outside of this component. Furthermore, support for not enrolled users is needed, using a temporary ID until the user is correctly identified.

1.1.6 Turn-Taking Module

1.1.6.1 Function.

This component decides whether the robot should take the turn and start talking, based on silence duration and the face orientation of the user currently speaking.

1.1.6.2 Pub: turn.

Publish information about the last text transcribed and whether the robot is taking the turn.

1.1.6.3 Sub: Transcription – transcribed.

When a user has finished talking the robot takes the turn if the current user is facing the robot, or after a long pause.

1.1.6.4 Sub: Speaker Awareness – user-angle.

Keeps track of the direction of arrival of the current user's voice.

1.1.6.5 Reflection.

At the moment the face orientation of the users is the main factor determining whether the robot takes the turn. In the future, additional factors such as the conversation contents could be taken into account to take this decision.

1.1.7 Conversation Manager Module

1.1.7.1 Function.

This component keeps track of the conversation and controls what the robot will say. It uses GPT-3.5 to generate the answer using the streaming mode, meaning that the server will send the response as a sequence of chunks as soon as available, instead of waiting for the full answer to be generated. In addition, the model is asked to identify the addressee of the response among the recognised users and to include all participants. The prompt used is reported in this appendix, under Section 1.3.

1.1.7.2 Pub: addressee.

Publish a new addressee when it is available.

1.1.7.3 Pub: text.

This component accumulates the responses streamed by the model. The response is published as soon as a sentence is complete – meaning that it ends with one of '.!?'.

1.1.7.4 Sub: Transcription – transcribed.

Sends the transcribed text to the model. In the prompt, the text is associated with the speaker's name. If the speaker has not been recognised yet by the Diarisation module, this waits an additional 800 milliseconds before using the ID from the speaker Transcription module, which is less reliable. Instead, if not even the Transcription module has recognised the speaker, this waits 2 seconds. If the user is still unrecognised, the prompt will contain the transcribed text under a generic label "user".

1.1.7.5 Sub: Interactions – spoken-text.

Adds the text spoken by the robot to the conversation history. The text is not added directly when it is generated, as one of the users might interrupt the robot. With this architecture, only the text that was actually spoken is added to the conversation.

1.1.7.6 Sub: Turn-Taking – turn

Obtain the information about whether the robot should take the turn.

1.1.7.7 Sub: Face Tracking – face-position, Diarisation – speaker.

Merges the speaker's information with the information on conversation participants.

1.1.7.8 Sub: Face Tracking – users.

Obtains information about the people recognised from the video stream and uses it to update the data on the conversation participants.

1.2 On Hardware Independence

Several of the modules reported above are strongly dependent on the hardware. Specifically, the Speaker Awareness and Interactions components depend respectively on the ReSpeaker microphone and the Furhat robot.

However, these do not actually rely on the specific hardware, only on the available capabilities. For instance, any microphone able to detect a sound's direction of arrival could be used with minor changes to the overall code structure. Similarly, the Furhat robot could be swapped with another provided a similar set of sensors and actuators are provided.

In addition, for testing purposes during the development phase, we implemented components that rely on a laptop's integrated microphone and camera. In these cases, the system continues to work, albeit with a lower performance.

1.3 The LLM Prompt

You are ⟨robot name⟩, a social robot. Engage in a warm, empathic, and casual chat with your friends. It is now ⟨date and time⟩ and we're speaking ⟨language⟩ in ⟨location⟩. Keep your answers concise in two to three sentences and ask engaging open questions. Feel free to express yourself naturally, like a human friend! Try to involve everyone in the conversation.

⟨conversation history⟩

Task: Social Robot Interaction. You are a social robot named ⟨robot name ⟩, engaging in a conversation with multiple people. You are permitted to lie, as long as you don't reveal yourself as an AI language model. Keep your answers brief and chatty (less than 2 sentences). Ask open questions to keep the conversation engaging, by involving all previous people, and showing interest. Adhere to social norms, the people are different individuals, do not mix up their information.

Input: Recognized people: ⟨users⟩

Action:

1: Chosen_person = A person from the list of Recognized people which you will address in the next sentence, based on previous conversation

2: Response = Generate a response based on the prompt and the selected person

Output result of your action in the following FORMAT (using Python f-string syntax): f"Addressee: {Chosen_person}; Response: {Response}"

2 QUESTIONNAIRES

To assess participants' perceptions of engagement, interaction quality, trust, and system performance, we administered a set of post-test questionnaires following each scenario. Some items were adapted from established scales (RoSAS, IQ, and MDMT) to fit the specific context of open-ended, multi-party

interaction. All items are reported in full below; no items were removed or selectively omitted. Open-ended prompts were included to capture detailed qualitative feedback.

2.1 Post-Test Scenario Questionnaires (Group and Parallel)

2.1.1 Engagement (RoSAS)

Q.1 To what extent did you find yourself engaged in the conversation with Furhat?
Q.2 How interesting did you find Furhat's contributions to the conversation?
Q.3 How well did Furhat's personality or demeanour fit the conversation topic?
Q.4 How motivated were you to participate actively in the conversation with Furhat?
Q.5 Did you feel a sense of social presence from Furhat during the conversation?
- If yes, please describe what aspects of the interaction contributed to this feeling.
- If no, what elements were missing that would have created a stronger sense of social presence?

2.1.2 User Experience (IQ)

Q.1 How natural did Furhat's conversation flow feel to you?
- Can you elaborate on what made the conversation flow feel natural or unnatural?

Q.2 Did Furhat provide coherent and contextually relevant contributions?
Q.3 Did Furhat maintain a consistent conversational style throughout the interaction?
Q.4 How enjoyable was your interaction with Furhat?
Q.5 How clear and easy to follow were Furhat's visual cues (e.g., eye gaze, body language)?
- Can you provide examples of Furhat's visual cues that were helpful or confusing?

2.1.3 Usability (MDMT)

Q.1 How easy was it to interact with Furhat (e.g., taking turns, asking questions)?
Q.2 Did you experience any technical difficulties during your interaction?
- If so, please describe.

Q.3 How intuitive was it to know when it was your turn to speak during the conversation?
- Can you suggest ways to improve the clarity of turn-taking cues for future interactions with Furhat?

2.1.4 Performance

Q.1 How well did Furhat understand your questions and requests?
Q.2 How relevant were Furhat's responses to the conversation topic?
Q.3 How accurate was Furhat's factual information or knowledge shared during the conversation?
Q.4 How well did Furhat recover from misunderstandings or errors in the conversation?
- Can you describe a specific situation where Furhat misunderstood something or made an error? How did Furhat attempt to recover?

Q.5 Overall, how enjoyable was your interaction with Furhat?

2.1.5 Performance (Turn-Taking)

Q.1 Did Furhat interrupt or speak over you or your partner?
- If yes, can you describe specific instances where this happened and how it affected the flow of the conversation?

Q.2 Did Furhat consistently identify the correct speaker (you or your partner) when responding to questions or comments?

- If no, describe the situation where this occurred.

Q.3 How effectively did Furhat use nonverbal cues to signal turn transitions?

Q.4 How appropriate was Furhat's timing when initiating turns to speak in the conversation?

2.2 Post-Test Overall Session Questionnaire

2.2.1 User Experience

Q.1 How easy was it to understand Furhat's responses?

- Were there any specific instances where Furhat's responses were difficult to understand? If so, please describe.

Q.2 Did Furhat's voice quality and tone enhance or detract from the conversation?

Q.3 How likeable or friendly did you find Furhat?

Q.4 How trustworthy did Furhat seem as a conversational partner?

- Why so?

Q.5 How intelligent or knowledgeable did Furhat appear to be?

Q.6 How human-like did Furhat's behaviour seem to you?

- Can you describe what aspects of Furhat's behaviour felt human-like and what aspects felt artificial?

2.2.2 Usability and Turn-Taking

Q.1 How easy was it to interact with Furhat (e.g., taking turns, asking questions)?

Q.2 Did you experience any technical difficulties during your interaction?

- If so, please describe.

Q.3 How intuitive was it to know when it was your turn to speak during the conversation?

- Can you suggest ways to improve the clarity of turn-taking cues for future interactions with Furhat?

Q.4 Was Furhat's response length appropriate to maintain a balanced and natural flow in the conversation?

- If not, what felt too short or too long?

2.2.3 Group Settings

Q.1 In the group conversation, did Furhat successfully follow the flow of the conversation between you and your partner?

Q.2 Did Furhat interject or contribute to the conversation in a natural way?

Q.3 Did Furhat provide responses or questions that effectively encouraged further discussion?

2.2.4 Parallel Settings

Q.1 In the separate conversation, did Furhat seem to be able to differentiate between you and your partner as separate individuals?

Q.2 Did Furhat seem to be able to separate the goal of both you and your partner?

Q.3 Did Furhat mistakenly involve the other person in your question (or the other way around)?

FIGURES

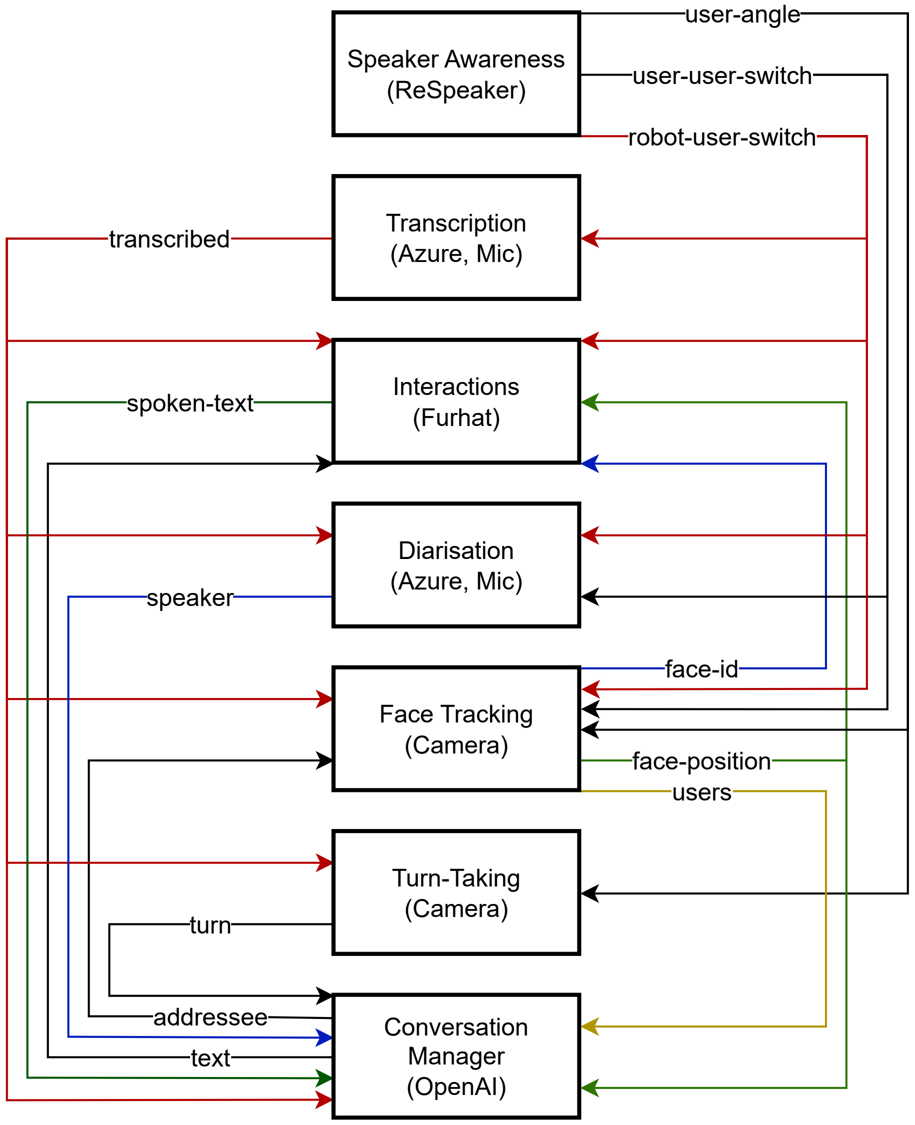

Figure S1. Overview of the system's components and their connections. External components are between parenthesis: the ReSpeaker microphone for direction of arrival, or "Mic" for the audio stream, the Furhat robot or the Camera feed from the robot, and the external services used. Colours are only to help distinguish between the lines.
